# Dark-adaptation in the eyes of a lake and a sea population of opossum shrimp (*Mysis relicta*): retinoid isomer dynamics, rhodopsin regeneration, and recovery of light sensitivity

**DOI:** 10.1007/s00359-020-01444-4

**Published:** 2020-09-03

**Authors:** Tatiana Feldman, Marina Yakovleva, Martta Viljanen, Magnus Lindström, Kristian Donner, Mikhail Ostrovsky

**Affiliations:** 1 Department of Molecular Physiology, Biological Faculty, Lomonosov Moscow State University, Leninskie Gory 1, Moscow, Russia 119991; 2 Emanuel Institute of Biochemical Physics, Russian Academy of Sciences, Kosygin st. 4, Moscow, Russia 119334; 3 Molecular and Integrative Biosciences Research Program, Faculty of Biological and Environmental Sciences, University of Helsinki, Helsinki, Finland; 4 Tvärminne Zoological Station, Faculty of Biological and Environmental Sciences, University of Helsinki, Helsinki, Finland

**Keywords:** Vision, Rhabdomeric photoreceptor, Visual cycle, Crustacea, Baltic Sea

## Abstract

We have studied dark-adaptation at three levels in the eyes of the crustacean *Mysis relicta* over 2–3 weeks after exposing initially dark-adapted animals to strong white light: regeneration of 11-*cis* retinal through the retinoid cycle (by HPLC), restoration of native rhodopsin in photoreceptor membranes (by MSP), and recovery of eye photosensitivity (by ERG). We compare two model populations (“Sea”, S_p_, and “Lake”, L_p_) inhabiting, respectively, a low light and an extremely dark environment. 11-*cis* retinal reached 60–70% of the pre-exposure levels after 2 weeks in darkness in both populations. The only significant L_p_/S_p_ difference in the retinoid cycle was that L_p_ had much higher levels of retinol, both basal and light-released. In S_p_, rhodopsin restoration and eye photoresponse recovery parallelled 11-*cis* retinal regeneration. In L_p_, however, even after 3 weeks only ca. 25% of the rhabdoms studied had incorporated new rhodopsin, and eye photosensitivity showed only incipient recovery from severe depression. The absorbance spectra of the majority of the L_p_ rhabdoms stayed constant around 490–500 nm, consistent with metarhodopsin II dominance. We conclude that sensitivity recovery of S_p_ eyes was rate-limited by the regeneration of 11-*cis* retinal, whilst that of L_p_ eyes was limited by inertia in photoreceptor membrane turnover.

## Introduction

All visual pigments (rhodopsins) consist of a G-protein-coupled receptor protein (opsin) covalently binding a light-sensitive cofactor (the chromophore), which is some form of retinal. The first event in vision is photoisomerization of the retinal from the 11-*cis* to the all-*trans* configuration, which triggers a sequence of very fast conformational changes of the opsin leading to the reasonably long-lived, G-protein-activating form metarhodopsin II (MII) (Fain et al. 2010). MII activity is terminated in steps by phosphorylation and arrestin binding. A necessary condition for complete recovery of photoreceptor light sensitivity in darkness is restoration of the full complement of native rhodopsin (R) with 11-*cis* retinal. In both vertebrates and arthropods, this involves enzymatic conversion of all-*trans* to 11-*cis* retinoid through several steps, partly in cells other than the photoreceptors (e.g., Wang et al. 2010; Kiser et al. 2012). In the c-opsins of the vertebrate ciliary photoreceptors (rods and cones), all-*trans* retinal soon detaches from MII, is reduced to retinol in the photoreceptor outer segment, and transported to the retinal pigment epithelium (RPE). There, all-*trans* retinol is converted to 11-*cis* retinol in an enzymatic reaction chain, whereupon the 11-*cis* retinol is oxidized to retinal and delivered over the interphotoreceptor matrix to free opsins in the photoreceptor cells. For cones, this visual cycle (or parts of it) may occur in Müller cells, or in the photoreceptors themselves, instead of the RPE (Wang and Kefalov 2011). Recently, it has been shown that visual-pigment renewal in mammalian photoreceptor membranes may also occur through a non-enzymatic, light-driven process involving retinyl phospholipids (Kaylor et al. 2017).

In the r-opsins of microvillar photoreceptors (such as the retinula cells of insects and crustaceans), a light-driven mechanism for fast regeneration of 11-*cis* retinal and native rhodopsin was long thought to be dominant or even exclusive (Hamdorf et al. 1973; Hillman et al. 1983; Schwemer 1984, 1989). The MII ↔ R system constitutes a bistable switch, as the MII–arrestin complex is thermally stable and the MII structure allows photoisomerization of all-*trans* back to 11-*cis* (Kiselev and Subramaniam 1994; Stavenga and Hardie 2011). At any illumination level, an R:MII equilibrium is established that depends on the wavelength composition of the light and the spectral absorbance of the respective R and MII pigments (Hamdorf 1979). Thus light sensitivity can be sustained continuously even in very bright light, but sensitivity recovery in darkness cannot, of course, be based on a light-driven mechanism. The first studies of “dark” mechanisms nearly 50 years ago were restricted to dim-light crustaceans like lobster and crayfish, but even then Timothy Goldsmith noted (Goldsmith 1975): “Photoregeneration of rhodopsin from metarhodopsin is likely an important mechanism of recovery in certain arthropods such as diurnal insects, but dark mechanisms of recovery also exist in all phyla”. In the present century, an enzymatic visual cycle involving eye pigment cells has been unravelled even in the (diurnal) insect model *par préference*, *Drosophila* (Wang et al. 2010; Montell 2012).

Even if both light-dependent and light-independent mechanisms may be present in all species, the relative weights of the two routes must differ depending on the amount of light typically available to the animal. Here, we study two populations of *Mysis relicta* from southern Finland, with a long history as a model pair for studies of evolutionary and epigenetic divergence in response to differing dim-light environments (Lindström and Nilsson 1988; Jokela-Määttä et al. 2005; Donner et al. 2016; Viljanen et al. 2017). They have been postglacially isolated in what are now a very dark brown freshwater lake (population denoted L_p_) and a somewhat less dark, greenish, oligohaline bay of the Baltic Sea (population denoted S_p_). At the depths where they usually dwell (> 20 m), the light intensity at the maximally transmitted wavelengths (ca 680 nm in the lake and 580 nm in the sea habitat) has dropped by at least 10 and 7 log units, respectively, compared with the surface (Donner et al. 2016). The spectral sensitivity maxima as measured by single-rhabdom absorption spectra lie at ca. 560 nm in L_p_ and 535 nm in S_p_ on average, a difference that can be explained by expression of two rhodopsins with *λ*_max_ around 525 and 570 nm in unequal proportions (Zak et al. 2013; Donner et al. 2016). On the other hand, measurements of post-bleach absorbance in single rhabdoms indicate that MII peaks around 490–500 nm in both populations (Viljanen et al. 2017), strengthening the conclusion that photoreconversion MII → R must be negligible in their long-wavelength-dominated, low-light habitats.

The two populations differ in their capacity to recover from strong light exposures, which, in L_p_ eyes, may cause long-term sensitivity suppression and disorganization of the photoreceptive membranes, often described as light damage (Lindström and Nilsson 1988; Lindström et al. 1988, 2001). On the other hand, Viljanen et al. (2017) have argued that these effects represent extremes on a continuum of physiological responses to varying light levels, and showed that slow acclimation of L_p_ to higher illumination levels over months mitigates or even abolishes the suppression of L_p_ light sensitivity upon strong exposures.

**Fig. 1:**
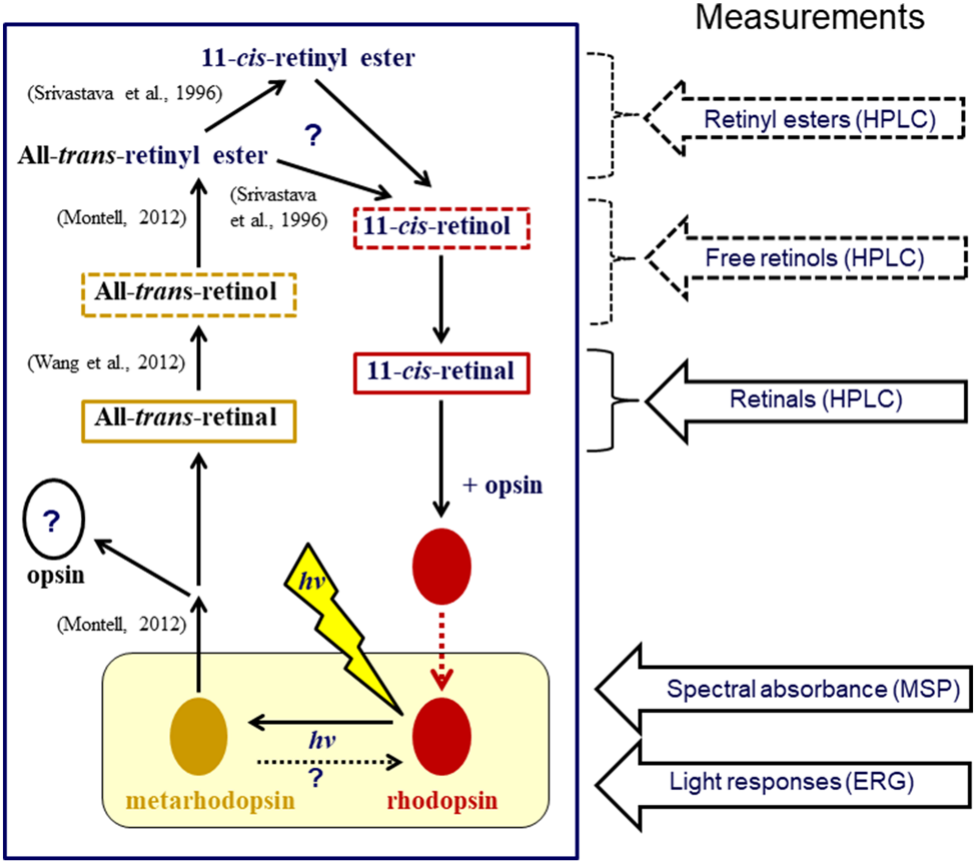
Hypothetical scheme for the visual cycle in the *Mysis* eye, compiled from a number of studies on crustaceans and insects. Some recent key references are indicated in the figure. We use this as a conceptual scaffold when measuring the dynamics and interpreting the meaning of changes at several levels during 2–3 weeks of dark-adaptation after exposing living, initially dark-adapted animals to a standard strong white light (lightning symbol). The different levels of the study are indicated by the arrows on the right, where the three uppermost measurements, based on HPLC and spectrophotometry, concern the retinoid cycle that underlies synthesis of 11-*cis* retinal for regeneration of rhodopsin. Events in the photoreceptor membranes are enclosed in the pale yellow box at the bottom, studied by microspectrophotometry (visual pigments) and electroretinography (light responsiveness of the eye). Direct reconversion of metarhodopsin to rhodopsin by light, as suggested by the dashed arrow at the bottom, is important in diurnal insects, but can play at most a minor role in *Mysis relicta*. The other question marks indicate other points not resolved by the present experiments. The scheme is based on a large body of work on the arthropod visual cycle conducted over more than half a century (see text for references)

The aim of this work was to analyze which mechanisms rate-limit the recovery of light sensitivity in the two populations over time scales of weeks in darkness after a standardized, strong white-light exposure. The conceptual background is the generalized scheme of the arthropod visual cycle shown in Fig. 1, compiled from the extensive literature on dark-adaptation in crustaceans and insects published over more than half a century (Goldsmith and Warner 1964; Goldsmith 1975; Barnes and Goldsmith 1977; Bruno et al. 1977; Cronin and Goldsmith 1984; Smith and Goldsmith 1991; Goldsmith and Cronin 1993; Donner et al. 1994; Srivastava et al. 1996, Srivastava and Goldsmith 1997; Wang et al. 2010, 2012a, b; Frank et al. 2012; Montell 2012). The arrows indicate the main parts of our study, all performed on the same cohorts of animals subjected to the same treatments. First, we measured the dynamics of changes in retinal, retinol, and retinyl ester isomers by high-performance liquid chromatography (HPLC) and spectrophotometry in eye extracts. Second, we measured changes in the spectral absorbance of single rhabdoms by microspectrophotometry (MSP), asking to what extent the kinetics of restoration of native rhodopsin in the photoreceptor membranes parallels regeneration of 11-*cis* retinal. Third, we measured the recovery of electrical light responses of whole eyes by electroretinography (ERG) to clarify how physiological function correlates with the two aforementioned processes.

## Materials and methods

### Animals

#### Capture and maintenance

The study animals were opossum shrimps (*Mysis relicta *sensu stricto, Väinölä 1986) from two populations, one living in the brackish-water Baltic Sea at the south-west coast of Finland, the other in a deep brown freshwater lake in southern Finland (coordinates N 59° 59.90′ E 23° 27.35′ and N 60° 00.09′ E 23° 27.52′, respectively). They will be denoted S_p_ and L_p_ (for Sea, Pojoviken and Lake, Pääjärvi) in line with our usage in the previous studies of these and other populations (Dontsov et al. 1999; Jokela-Määttä et al. 2005; Feldman et al. 2010; Zak et al. 2013; Donner et al. 2016; Viljanen et al. 2017). The S_p_ animals were caught in daytime from a depth of about 20 m with an epibenthic sledge ending in a plastic bag. The L_p_ animals were caught from a depth of 60–75 m by a vertical net ending in a jar, also in daytime, but care was taken to protect them from strong light exposures. The animals were immediately transferred to plastic bags containing water collected at the same time from the same depth, enclosed in Styrofoam boxes. They were transported in dark containers in oxygen-rich water held at the temperature of the sampling locality to Tvärminne Zoological Station (University of Helsinki), where, as a rule, they were kept in dark-aquaria at 4–9 °C for at least 1 month before experiments started. There was one exception, where a batch of L_p_ animals that had apparently experienced significant light exposure during capture was (due to logistic constraints) dark-adapted for only 1 week before the first MSP measurements, with the result that the eyes were in a bleached condition at the outset (the MSP series shown as triangles in Fig. 5b).

#### Ethical statement

*Mysis relicta* is not an endangered species, but on the contrary, the most common macrocrustacean in Finnish waters. Under Finnish legislation, no permit is needed for the sampling of invertebrates. The S_p_ sampling area (Pojoviken Bay) is a public shipping lane operated by the Finnish Transport Agency. Scientific study is part of the governance of the area, i.e., sampling for scientific purposes is not only allowed, but part of the intended use of the area. The L_p_ sampling area (Lake Pääjärvi) is a public water body, which has served as a limnologic study area of the University of Helsinki since 1953. Quite regardless of any the aforementioned circumstances, Finnish legislation guarantees public access according to the general principle of “everyman’s right” (common rights) to all areas regardless of ownership (private/state/municipal), unless explicit and precisely specified regulations apply (which is not the case here). Common rights include unrestricted sampling for scientific purposes of such invertebrate species that are not defined as endangered. The land-owner’s permission is never required for these purposes.

#### Standard light exposure

All animals except the dark-adapted controls were first subjected to a standardized exposure to strong white light. They were placed in a white bucket in an external container with water and ice, and illuminated for 30 min by a ring lamp placed 15 cm above the water. The light had a pseudo-white spectrum spanning approximately 400–700 nm (see Fig. 2e in Viljanen et al. 2017) and intensity ~ 10^12^ photons m^−2^ s^−1^ nm^−1^ measured at the water surface. The percentage of native R remaining after this exposure cannot be very precisely estimated due to the unknown position of screening pigments, which, in their light-adapted position, may filter > 90% of incoming light (Jokela-Määttä et al. 2005), the varying orientation and position of ommatidia, and the unknown degree of photoreconversion MII → R. Under the conservative assumption that the light intensity incident on the photoreceptive membranes is only 1% of that at the water surface, we estimate that more than 99.5% of all rhodopsins have at least initially been converted to MII during the 30 min exposure. It is also worth noting that the effects of such a strong exposure are likely to be fairly homogeneous throughout the eye.

**Fig. 2:**
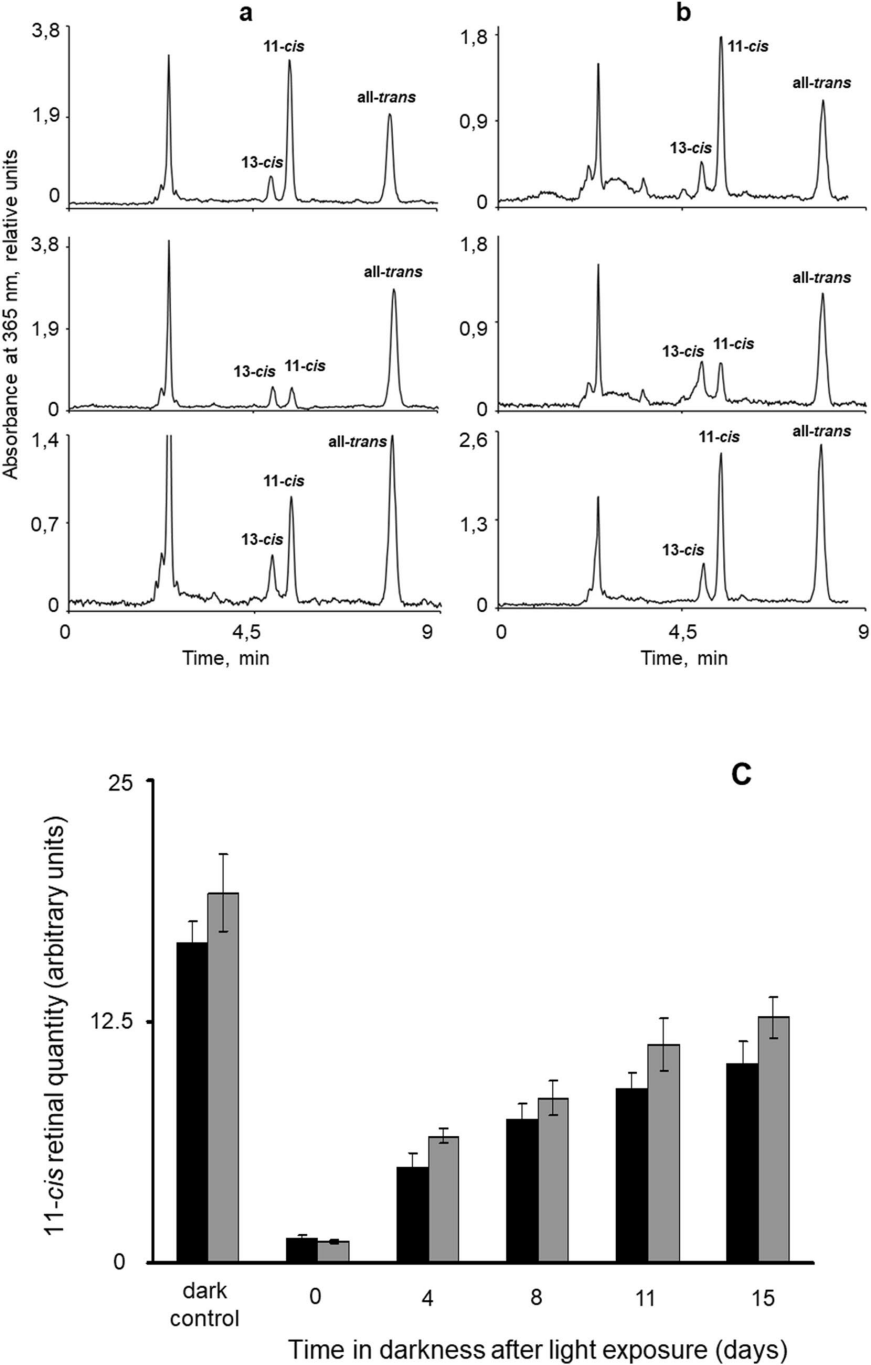
HPLC analysis of hexane extracts from the eyes of *Mysis relicta*. **a** and **b** Original chromatograms: **a** from L_p_ and **b** from S_p_ eyes. Top row: “dark” controls, i.e., samples from animals that had been kept in continuous darkness for 42 days (L_p_) and 58 days (S_p_) after capture; middle row: samples prepared immediately after the standard exposure to strong white light; bottom row: samples from animals that had been dark-adapted for 15 days after the exposure. Absorbance was measured at 365 nm. **c** Amounts (arbitrary units, Table 2) of 11-*cis* retinal in L_p_ (black) and S_p_ (grey) eyes in dark-adapted controls and at different times spent in darkness after the standard light exposure

Immediately after the illumination, a first sample was taken for preparation for “day 0” post-exposure measurements. The rest of the animals were returned to the dark-aquaria and sampled at predefined intervals between 4 and 20 days after the exposure. Non-exposed animals that had been kept continuously in darkness for at least 1 month served as controls. The light exposures were done at Tvärminne Zoological Station, where the animals were maintained, where ERG experiments were done, and where deep-frozen specimens for biochemistry in Moscow were prepared. For MSP, however, the light-exposed, living animals had to be transported from Tvärminne to Helsinki, and for that reason, “day 0” data are missing in the present sets of MSP measurements (Figs. 5 and 6).

### Measurement of retinoid dynamics

#### Chemicals

The reagents were of biochemical or reagent grade purity, obtained from Sigma-Aldrich (USA) and Component-Reactive Ltd (Russia). Solvents hexane, dichloromethane, ethyl acetate, and methanol of HPLC-grade purity were purchased from Biosolve (The Netherlands). All*-trans* retinal (A1) was obtained from Sigma-Aldrich (USA). All*-trans*, 11-*cis* and 13-*cis* isomers of retinol were obtained from Sigma (USA).

#### Preparation of samples

Immediately after the standard light exposure (see above), the first samples of both S_p_ and L_p_ animals were prepared. Twenty animals per sample were decapitated and a small part of the head with eyes and stalks but without antennas were cut out under IR light. The heads were put into Eppendorf tubes cooled by an ice bath, and when all heads had been collected, the tubes were put in the deep freeze at − 85 ºC. All this was done in darkness in Tvärminne. The deep-frozen and light-shielded samples were transported by train to Moscow, where the following steps were carried out in a room lit only by dim red light. The eye samples were thoroughly homogenized in 2 mL of distilled water. The homogenate was transferred to a round-bottom flask and treated 2 min with 1 mL formaldehyde. Following addition of 2 mL of dichloromethane the suspension was incubated for 10 min at 4 °C, after which 2 mL hexane was added. The mixture was then centrifuged (680*g*, 10 min, 4 °C). The upper hexane phase was drawn into a syringe and transferred to a flask. The extraction was repeated twice, and the organic layers were combined. The hexane extract was evaporated using a vacuum pump (Vacuubrand MZ 2CNT + AK + M + D, Germany). Each dried sample was resuspended in 1 mL hexane for the recording of absorption spectra. After that, the solvent was removed under reduced pressure. The dried extract was dissolved in 100 μL of hexane for subsequent HPLC analysis.

#### Spectrophotometry

Absorption spectra of the hexane extracts were recorded on a Shimadzu UV-1700 spectrophotometer (Japan). We have previously found that the shape and intensity of the carotenoid bands (400–550 nm) in the hexane extract spectra are practically identical in samples prepared from the same amount of animals from the two populations (Feldman et al. 2010). For comparative analysis of the absolute amounts of retinoids in the eyes, the spectra were, therefore, normalized to the same value at 480 nm.

#### Analysis of retinoid isomers

*Retinal* Analysis was performed on an HPLC system Knauer (Germany) with detector K-2501 with variable wavelength. The detection wavelength used was 365 nm. Optimal analytical separation of retinal isomers was achieved with column Silica (5 μm, 250 × 4.5 mm, IBM Instruments, USA), using as eluent hexane–ethyl acetate (95%—5% v/v) and flow rate 1.5 mL/min. To calculate the content of retinal isomers in the samples studied, external standards of 13-*cis* and all-t*rans* retinal isomers (Sigma, USA) were used. The peak position of 11-*cis* retinal was determined according to the analysis in Belikov et al. (2014). The relative content of each component (*i*) in the mixture is basically the surface area (integral) under the peak corresponding to that component (*S*_*i*_) taken as a percentage of the summed surface areas of all peaks (Σ *S*_i_). However, to translate peak sizes into concentrations, it is necessary to correct for the differences in the extinction coefficients *E* [L mol^−1^ cm^−1^] of the components at the detection wavelength (365 nm). The values used were 38,800 for 13-*cis* (Garwin 2000), 26,400 for 11-*cis* (Yoshizawa 1984), and 44,300 for all-*trans* retinal (Hubbard 1971). Thus, the percentage of component *i* was calculated as 100 × (*S*_*i*_*/E*_*i*_) / Σ (*S*_*i*_*/E*_*i*_). The values given in the “Results” section are means ± SEMs of measurements from three chromatograms separately recorded from each sample.

*Retinol* Retinols were analyzed as described above for retinals, but using the detection wavelength 325 nm and hexane–ethanol–diethyl ether (92%–1%–7% v/v) as eluent and flow rate 1.0 mL/min. External standards of 13-*cis*, 11-*cis* and all-t*rans* retinol isomers were used for calibration. The values used for the extinction coefficients *E* [L mol^−1^ cm^−1^] at 325 nm were 48,305 for 13-*cis* (Kuksa et al. 2003), 34,100 for 11-*cis*, and 52,100 for all-*trans* retinol isomers (Landers 1987). The relative contents of the different components were calculated as described above for retinals.

*Retinyl esters* Retinyl esters were analyzed as described by Goldsmith and Cronin (1993). Samples were dried and saponified in 3% KOH in methanol for 30 min at 30 °C. An equal volume of water was added, the retinoids were extracted into hexane, and the hexane was washed several times with water to remove traces of base. Thus, the sample was divided into two equal parts. Part 1 was immediately analyzed by HPLC for free retinols as described above, while part 2 was subjected to saponification before HPLC analysis. To obtain the content of retinyl esters, the values for free retinols from part 1 were subtracted from the values from part 2.

*Conversion into measures proportional to absolute quantities* The contents of each retinoid (*C*_*i*_) can be quantitatively compared between samples through normalization of each by factors that make the absorption spectra of the respective total hexane extracts coincide at the invariant carotenoid peak 480 nm (Feldman et al. 2010; see "Spectrophotometry" above). This does not, of course, give absolute values, but allows the quantification of the dynamics of change in retinoid isomers from samples taken at different time points. Thus, for this purpose, the scaled surface area of each component (*S*_*i*_/*E*_*i*_) was divided by the normalization factor at 480 nm for the total-extract absorption spectrum of the respective sample (*K*_*n*_), where *n* is the sample number. This was applied both to the retinal and the retinol analyses according to the formula:

1 $$C_{i,n} = S_{i,n} /(E_{i} \times K_{n} ).$$

### Measurement and analysis of absorption spectra of single rhabdoms by MSP

Absorption spectra from single rhabdoms were measured with a single-beam, fast wavelength-scanning microspectrophotometer as described by Govardovskii et al. (2000), Donner et al. (2016) and Viljanen et al. (2017). All handling and preparation took place in darkness under IR viewing. Since rhabdoms of animals that had been subjected to the standard light exposure were often broken and/or covered with dark screening pigment, the measuring beam was sometimes fitted to pieces of rhabdoms instead of intact rhabdoms. Absorption spectra were recorded from 15–25 rhabdoms per individual, depending on the quality of the sample, and 2–9 individuals per time point (4, 8, 12 and 16 days, in L_p_ also 20 days, after the exposure). The recording beam was linearly polarized and spectra were recorded at each site with both transversal (T) and longitudinal (L) polarization relative to the long axis of the rhabdom. Wavelengths of maximum absorption (*λ*_max_) were extracted from the same data set in three different ways:

1. The single-rhabdom spectra were averaged within each individual, and the A2 template of Govardovskii et al. (2000) was manually fitted to the averaged spectra after zero-line correction for possible drift. Although *Mysis relicta* use only A1 chromophore (Belikov et al. 2014), the A1 pigment template is too narrow to fit the single-rhabdom spectra, which are constituted by the summed absorbance of two pigments (Zak et al. 2013; Donner et al. 2016). By contrast, the broader A2 pigment template provides fair fits to these composite spectra at least in the dark-adapted state, allowing their shape and position to be captured in a single parameter (*λ*_max_). For purely descriptive purposes, this is preferable to fitting sums of two A1 templates, which requires fine-tuning of three parameters (two *λ*_max_ values, and the relative weight of the two templates, see Donner et al. (2016)). After strong light exposures, however, a third spectral component due to metarhodopsins peaking at 490–500 nm emerges, and template fitting is not a useful way of characterizing these complex and often noisy spectra. Instead, we used the more direct methods (2) and (3).
2. The wavelength of peak absorption (*λ*_max_) was read automatically in Matlab from each smoothed single-rhabdom spectrum, after rejection of spectra of poor quality.
3. T-L difference spectra were computed for each rhabdom and *λ*_max_ was read from each difference spectrum automatically in Matlab. This method is particularly informative. Visual pigments in the membranes of the retinula cells are oriented predominantly with the chromophore along the microvillar axis, thus absorbing light polarized transversely to the long axis of the rhabdom (T) better than light polarized orthogonally to this (L). By contrast, absorption due to screening pigments or disorganized visual pigments, or spurious “absorption” due to scattering, exhibits no dichroism. Hence, T-L difference spectra purify absorption by visual pigments residing in rhabdomal membranes that retain some degree of microvillar organization, and eliminate absorption from other sources, including “diffuse” absorption by visual pigments (cf. Fig. 6c, d).

### Measurement of eye photoresponsiveness by ERG

Stimulus-intensity vs. response-amplitude data were recorded from intact eyes of isolated *Mysis* heads. Details of preparation, recording, light stimulation, light calibration, and data analysis have been described, with emphasis on different aspects, in Lindström and Nilsson (1988), Lindström et al. (1988), Pahlberg et al. (2015), Donner et al. (2016), and Viljanen et al. (2017), and will not be repeated here.

Eye photoresponsiveness was characterized by two parameters, the maximum (saturating) response amplitude (*U*_max_, mV) and the light intensity (in relative units) required to elicit a response with half of that amplitude (*I*_½_). Fractional sensitivity *S*, i.e., the fraction (or percentage) of *U*_max_ elicited per photoisomerization, is inversely proportional to *I*_½_ (*S* ∝ *I*_½_^−1^). Values of these parameters were determined by fitting the data with the Naka–Rushton modification of the Michaelis–Menten function:

2 $$U/U_{{\max }} = I^{n} /(I^{n} + I_{{1/2}} ^{n} ) ,$$

where *U* is response amplitude and *I* is stimulus intensity. Although truly saturated responses could not be recorded owing to the limitation of the light source, fitting Eq. 2 to the data allowed reasonably accurate determination of *U*_max_. The equation contains a third parameter (*n*), defining the steepness of the curve, and there is a certain degree of interdependence between the three parameters in fitting, but the uncertainty which it introduces in *U*_max_ and *I*_½_ is insignificant for our present purposes (cf. Viljanen et al. 2017). Fitting was done by iteration in Matlab.

## Results

### Retinoid dynamics during dark-adaptation

#### Retinal isomers

Retinal isomers 11-*cis*, all-*trans* and 13-*cis* were analyzed by HPLC in extracts from small pieces of head including the eyes but not antennas. Examples of chromatograms recorded with detection wavelength 365 nm are shown in Fig. 2a (L_p_) and b (S_p_) with 13-*cis*, 11-*cis* and all-*trans* peaks labelled as originally identified against standards. The areas under the peaks corresponding to each of the three isomers can be measured and recalculated to percentages (Table 1) as well as to values proportional to the absolute amounts of each isomer (Table 2) (Materials and methods, Eq. 1).

**Table 1:** Percentages of 13-*cis*, 11-*cis* and all-*trans* isomers out of total retinal in the eyes of S_p_ and L_p_ animals: non-exposed dark-adapted animals and animals that have spent different times in darkness after the standard strong light exposure

Animals	*N*	Sample: days of dark-adaptation of the animals	Retinal isomers %		
			13-*cis*	11-*cis*	All-*trans*
Lake population (L_p_)	1	Non-exposed animals, 42 days of dark-adaptation after catching	6.59 ± 0.43	59.98 ± 0.76	33.43 ± 1.19
	2	Sample prepared immediately after the light exposure	9.44 ± 0.91	8.21 ± 0.78	82.41 ± 2.31
	3	4 days after exposure	12.06 ± 1.02	26.22 ± 1.68	61.72 ± 2.01
	4	8 days after exposure	9.97 ± 0.27	31.84 ± 1.31	58.18 ± 2.36
	5	11 days after exposure	11.21 ± 0.92	37.32 ± 1.57	51.47 ± 2.19
	6	15 days after exposure	9.65 ± 0.37	40.31 ± 0.89	50.05 ± 1.26
Sea population (S_p_)	1	Non-exposed animals, 58 days of dark-adaptation after catching	6.22 ± 0.32	65.33 ± 2.29	28.45 ± 1.01
	2	Sample prepared immediately after the light exposure	15.38 ± 1.09	6.32 ± 0.68	78.32 ± 2.23
	3	4 days after exposure	9.94 ± 0.93	38.25 ± 1.76	51.81 ± 3.69
	4	8 days after exposure	11.94 ± 1.06	46.31 ± 1.54	41.75 ± 2.17
	5	11 days after exposure	9.03 ± 0.58	48.21 ± 1.76	42.76 ± 1.67
	6	15 days after exposure	8.28 ± 1.08	50.34 ± 1.91	41.38 ± 1.33

**Table 2:** Amounts of 13-*cis*, 11-*cis* and all-*trans* retinal in the eyes of S_p_ and L_p_ animals: non-exposed dark-adapted animals and animals that have spent different times in darkness after the standard strong light exposure

Animals	*N*	Sample: days of dark-adaptation of the animals	Retinal isomers *C*_***i***_ × 10^6^		
			13-*cis*	11-*cis*	All-*trans*
Lake population (L_p_)	1	Non-exposed animals, 42 days of dark-adaptation after catching	1.82 ± 0.32	16.56 ± 1.12	9.24 ± 0.98
	2	Sample prepared immediately after the light exposure	1.43 ± 0.12	1.25 ± 0.23	12.51 ± 1.05
	3	4 days after exposure	2.27 ± 0.31	4.95 ± 0.76	11.66 ± 1.11
	4	8 days after exposure	2.32 ± 0.72	7.41 ± 0.83	13.54 ± 1.08
	5	11 days after exposure	2.69 ± 0.54	8.98 ± 0.91	12.39 ± 1.43
	6	15 days after exposure	2.47 ± 0.45	10.31 ± 1.14	12.80 ± 1.22
Sea population (S_p_)	1	Non-exposed animals, 58 days of dark-adaptation after catching	1.83 ± 0.27	19.17 ± 2.01	8.35 ± 0.76
	2	Sample prepared immediately after the light exposure	2.64 ± 0.38	1.08 ± 0.07	13.42 ± 1.05
	3	4 days after exposure	1.71 ± 0.19	6.56 ± 0.39	8.89 ± 0.67
	4	8 days after exposure	2.20 ± 0.22	8.53 ± 0.91	7.69 ± 0.96
	5	11 days after exposure	2.12 ± 0.41	11.31 ± 1.37	10.03 ± 1.03
	6	15 days after exposure	2.09 ± 0.36	12.72 ± 1.05	10.45 ± 0.99

The values are in arbitrary units proportional to the absolute quantities (see "Materials and methods", Eq. 1)

Control animals that had been kept in complete darkness for at least 1 month and not subjected to any light exposure did not differ significantly between the populations in the percentages of 11-*cis* and all-*trans* retinal (65% and 28% for S_p_; 60% and 33% for L_p_). The fact that such a large proportion of the retinal is in all-*trans* form suggests that 1 month in darkness is not enough for complete recovery from the light exposures associated with daytime catching (see "Materials and methods").

The values obtained immediately after the light exposure were also similar: the percentage of 11-*cis* out of total retinal decreased dramatically in both, to 6% (S_p_) and 8% (L_p_), and all-*trans* retinal increased to 78% (S_p_) and 82% (L_p_). The remaining fraction was 13-*cis* retinal, which increased somewhat. Thus, the initial conditions in darkness as well as immediately after the light exposure were practically identical in the eyes of S_p_ and L_p_ animals.

“Dark” regeneration of 11-*cis* retinal, as well as changes in 13-*cis* and all-*trans*, was monitored by measurements in samples from animals that had been left to dark-adapt in aquaria at ca. 9 ºC after the initial light exposure. Extracts were prepared and measurements done at four post-exposure time points (4, 8, 11, and 15 days). Table 1 shows the percentages of the three isomers. The rise in the percentage of 11-*cis* was somewhat faster in S_p_ than in L_p_, but the difference is moderate.

Measures proportional to the absolute amounts of the three isomers are presented in Table 2. The increase of 11-*cis* during dark-adaptation is graphically summarized for both populations in Fig. 2c. As already noted for the percentages, 11-*cis* regeneration was slightly but not dramatically faster in S_p_ than L_p_ animals. Neither population reached the initial, pre-exposure amounts within the time span of the experiment, 15 days. At that point, the amounts corresponded to 66% (S_p_) and 62% (L_p_) of the initial values. Note, however, that even in the initial, pre-exposure situation (animals that had been held in darkness for 42–58 days after capture), 11-*cis* constituted only 65% (S_p_) and 60% (L_p_) of the total retinal pool (Table 1).

The changes in total retinal in Table 2 are especially interesting. Comparing post-exposure day 0 with dark-adapted controls, decreases of 18 (S_p_) and 15 units (L_p_) in 11-*cis* are coupled to all-*trans* increases of only 5 (S_p_) and 3 units (L_p_), reducing total retinal by 40–45% in both populations. This implies that the all-*trans* retinal arising from the photoisomerization of 11-*cis* is quickly removed, presumably by reduction to all-*trans* retinol (cf. Fig. 1). During the subsequent course of dark-adaptation, the amounts of all-*trans* and 13-*cis* retinal change very little in both S_p_ and L_p_, and the slow increase in total retinal (reaching ca 90% of the control value after 15 days of dark-adaptation) depends entirely on the regeneration of 11-*cis*. It is worth noting, however, that even after 15 days, all-*trans* remains higher than 11-*cis* in L_p_ (12.8 vs. 10.3 units), whilst the opposite is true in S_p_ (10.5 vs. 12.7 units). This is likely to be associated with an inert store of MII in L_p_ membranes (see below).

#### Retinol isomers

Above, the post-exposure decrease in total retinal by more than 40% was tentatively attributed to fast reduction of all-*trans* retinal to all-*trans* retinol. On the opposite side of the visual cycle, 11-*cis* retinol is a precursor of 11-*cis* retinal (Goldsmith and Cronin 1993; Kuksa et al. 2003) (cf. Fig. 1). Our next task was to measure changes in retinol and its isomers in the same conditions as the changes in retinal isomers.

**Fig. 3:**
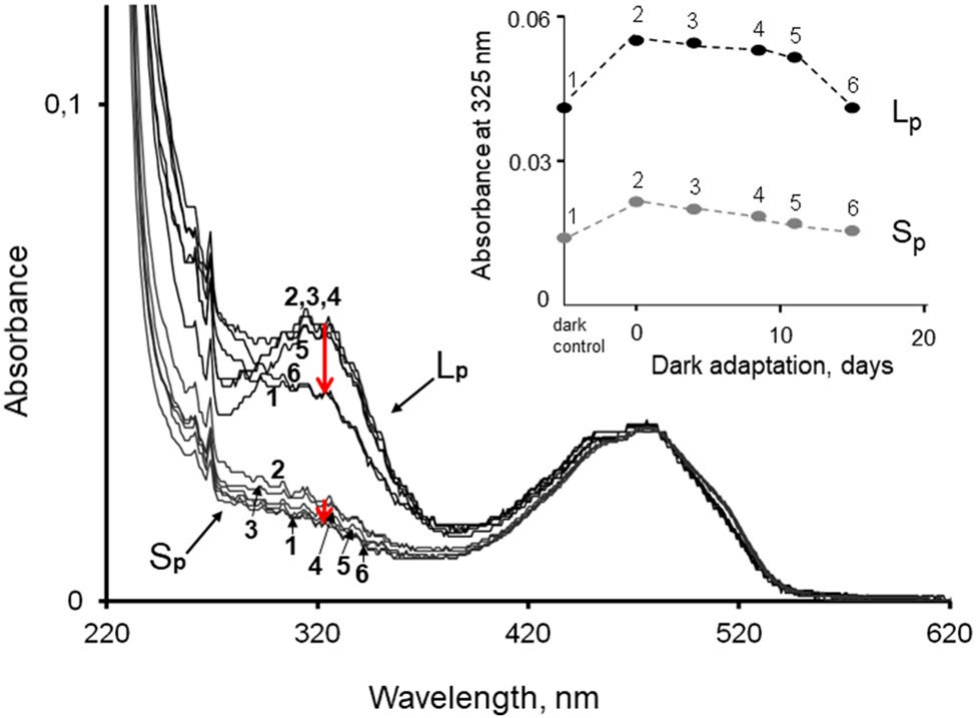
Spectral analysis of free retinol content in L_p_ and S_p_ eyes before and at different times after the standard light exposure. The curves of the Main panel show absorption spectra of total hexane extracts (the same extracts that were used for the HPLC analysis of retinals summarized in Tables 1 and 2). All the curves have been normalized to the same value at the at the invariant carotenoid peak 480 nm, which enables strict quantitative comparison between them (Materials and methods, Eq. 1). The upper family of six numbered curves are from L_p_ animals, the lower curve family from S_p_ animals. The curves marked (1) represent non-exposed, dark-adapted controls, the curves marked (2)–(6) represent animals that had spent, respectively, 0, 4, 8, 11, and 15 days in darkness after the light exposure. Note that in both L_p_ and S_p_, curve (6) practically coincides with the pre-exposure curve (1). The red arrows highlight the decreases in absorption in the region of 325 nm during dark-adaptation of animals after the exposure. The Inset shows the absorbance values measured at that wavelength, i.e., at the retinol absorbance peak, before and at different times after the light exposure, numbered as the corresponding curves in the main panel

Figure 3 shows changes in absorption spectra of total hexane extracts from the eyes of L_p_ animals (top family of curves) and S_p_ animals (bottom family of curves). These spectra are from the same samples used for analysis of retinals described in the previous section. There are two main bands: 380–550 nm, corresponding to carotenoids, and 270–380 nm corresponding to retinoids, where retinol (maximum absorption at 325 nm) is the dominant component, accounting for ca. 40% (Feldman et al. 2010).

The numbering of the curves (1–6) corresponds to eye extracts from animals in consecutive states of adaptation: curve (1) from dark-adapted animals that had not been exposed to light, curve (2) from animals sacrificed immediately after the bright-light exposure, and curves (3–6) from animals that had spent 4, 8, 11, and 15 days in darkness after the exposure. The red arrows highlight changes in absorbance around 325 nm, mainly representing retinol (cf. Feldman et al. 2010). As the curves are difficult to resolve by eye, the absorbance changes at this wavelength are plotted separately in the Insert. In both populations, the light exposure increased 325-nm absorbance markedly, but the increase was much greater in L_p_ (in absolute terms, although not in %), where the general level was 2-3-fold higher than in S_p_ throughout. Subsequent dark-adaptation was associated with a monotonic decrease of 325-nm absorbance, and after 15 days, it had returned to pre-exposure values.

In the following, the analysis of retinol isomer identity was restricted to L_p_ animals, where changes are larger than in S_p_. Furthermore, the data presented on L_p_ retinol isomers in Table 3 are from a different set of experiments than the data hitherto considered. The extraction protocol in this set was designed to enable parallel determination of retinyl esters by subtraction of free retinol from total retinol measured in aliquots of the same sample (see Materials and methods).

**Table 3:** Amounts of 13-*cis*, 11-*cis* and all-*trans* isomers of free retinol in the eyes of non-exposed dark-adapted animals and animals that have spent different times in darkness after the standard strong light exposure

Samples L_p_ animals	*N*	Days of dark-adaptation	Retinol isomers *C*_*i*_ × 10^6^		
			13-*cis*	11-*cis*	All*-trans*
Non-exposed dark-adapted animals	1	45	4.11 ± 0.46	25.25 ± 4.21	21.34 ± 1.12
Samples obtained at different times after the standard light exposure	2	0	19.25 ± 1.12	189.41 ± 5.67	86.41 ± 8.32
	3	4	12.93 ± 1.11	106.02 ± 6.45	57.05 ± 2.46
	4	8	10.87 ± 0.98	99.30 ± 1.41	58.42 ± 3.12
	5	12	10.21 ± 0.99	39.81 ± 2.02	39.83 ± 1.23

The values are in arbitrary units proportional to the absolute quantities (see "Materials and methods", Eq. 1)

**Fig. 4:**
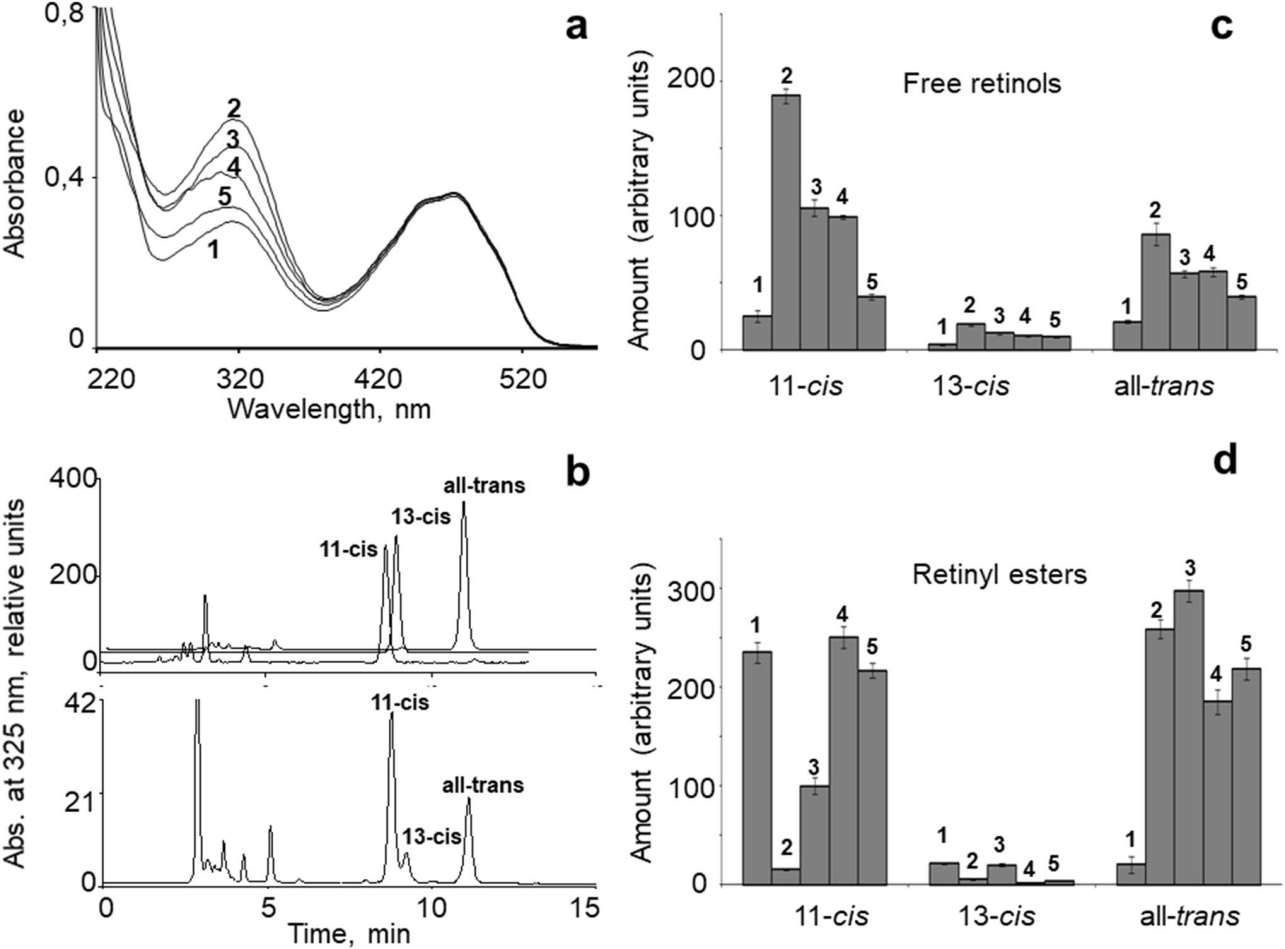
Spectral and HPLC analysis of retinol and retinyl ester isomers in L_p_ eyes before and at different times following the standard exposure to strong white light. **a** Absorption spectra of total hexane extracts: (1) in dark-adapted controls (non-exposed animals dark-adapted for 45 days after catching); (2) in extract prepared immediately after the exposure, (3–5) in extracts prepared from animals allowed to dark-adapt for 4, 8, and 12 days after the exposure. **b** HPLC analysis of hexane extracts. Top: chromatograms of retinol standards (11-*cis*, 13-*cis,* and all-*trans*). Bottom: sample from animal dark-adapted for 12 days after exposure, shown as an example. Absorbance was measured at 325 nm. **c** Amounts of the isomers of free retinol in samples prepared at time points encoded (1–5) as in panel (**a**) (Table 3). **d** Amounts of the isomers of retinyl esters, obtained by subtracting free retinols from total retinols measured in the same samples (Table 4). The *y*-axis gives the area under the respective peak of the chromatogram, scaled according to Eq. (1)

Figure 4 exemplifies recordings (a-b) and shows results (c-d) from this set of experiments. Panel a shows absorption spectra of total hexane extracts prepared in consecutive states of adaptation: from non-exposed animals (curve 1) and from animals sampled at different times after the light exposure (curves 2–5). These curves are similar to the retinol curves in Fig. 3, showing a strong light-induced increase followed by a return towards baseline over (in this case) 12 days. Panel b illustrates HPLC identification of the peaks (11-*cis*, 13-*cis,* and all-*trans*) in one sample (bottom chromatogram) based on comparison with retinol standards (top chromatograms), all recorded with the detection wavelength 325 nm. The areas under the peaks were measured and scaled as described in "Materials and methods" (Eq. 1) to yield the amounts of the respective retinol isomers.

As seen in Table 3, the amounts of free 11-*cis* and all-*trans* retinols were approximately equal in the eyes of dark-adapted animals. Interestingly, the light-induced retinol increase seen in the total-extract absorption spectra (panel a) involved strong increases in *both* the main isomers: 11-*cis* increased by as much as 7.5-fold, all-*trans* by fourfold. This is consistent with the literature on crustaceans and insects (Goldsmith and Cronin 1993; Wakakuwa et al. 2003), suggesting that light-dependent release of retinol from large reserves may be a widespread feature of the retinoid cycle in arthropods. After the initial increase, all isomers of free retinol decreased steadily towards the initial pre-exposure levels (post-exposure days 4–12, numbered 2–5). These changes are graphically summarized in Fig. 4c.

#### Retinyl ester isomers

Retinyl esters constitute the third main component of the retinoid cycle (Fig. 1). Amounts of the isomers of retinyl esters in L_p_ eyes were determined by subtraction of free retinols from total retinols measured from aliquots of the same sample. The amounts (same arbitrary units as in Table 3) are given in Table 4 and graphically summarized in Fig. 4d. In dark-adapted animals, most of the retinyl esters (approximately 85% of all) were in 11-*cis* form. The light exposure caused the level of 11-*cis* to drop sharply, to about 6% of the total, mirroring the dramatic increase in free 11-*cis* retinol seen in Fig. 4c. Thus, almost all 11-*cis* retinyl esters were hydrolyzed to free 11-*cis* retinol.

**Table 4:** Amounts of 13-*cis*, 11-*cis* and all-*trans* isomers of retinyl esters in the eyes of non-exposed dark-adapted animals and animals that have spent different times in darkness after the standard strong light exposure

Samples L_p_ animals	*N*	Days of dark-adaptation	Retinyl ester isomers *C*_*i*_ × 10^6^		
			13-*cis*	11-*cis*	All*-trans*
Non-exposed dark-adapted animals	1	45	21.62 ± 1.31	235.21 ± 10.22	20.21 ± 8.23
Samples obtained at different times after the standard light exposure	2	0	5.14 ± 0.83	15.56 ± 1.22	258.96 ± 9.38
	3	4	20.01 ± 1.11	99.77 ± 8.35	297.52 ± 11.26
	4	8	2.06 ± 0.86	250.31 ± 11.15	185.09 ± 12.32
	5	12	3.99 ± 0.56	216.81 ± 7.31	218.19 ± 10.91

The values are in arbitrary units proportional to the absolute quantities (see "Materials and methods", Eq. 1)

The proportion of all-*trans* retinyl esters, on the other hand, was insignificant in dark-adapted animals (about 8%), but jumped by more than tenfold immediately after the bright-light exposure (Fig. 4d, Table 4). A likely partial explanation is that this reflects light-induced release from the visual pigment of all-*trans* retinal, which is quickly reduced to all-*trans* retinol and converted into all-*trans* retinyl esters. It is worth noting that retinyl esters and retinol absorb in the same spectral region (around 325 nm); thus, the all-*trans* retinyl esters cannot have arisen from 11-*cis* → all-*trans* isomerization by our white light (which consists of wavelengths >  ~ 400 nm). During dark-adaptation, the amount of 11-*cis* retinyl esters grew back to its dark-adapted value in about a week (Fig. 4 d). Interestingly, the content of all-*trans* retinyl esters stayed more or less constant on its elevated post-exposure level over the 12 days of the experiment. This suggests a dynamic steadystate between the rate of enzymatic isomerization to 11-*cis* retinyl esters and the rate of synthesis from free all-*trans* retinol.

### Incorporation of newly synthesized rhodopsin into photoreceptor membranes

**Fig. 5:**
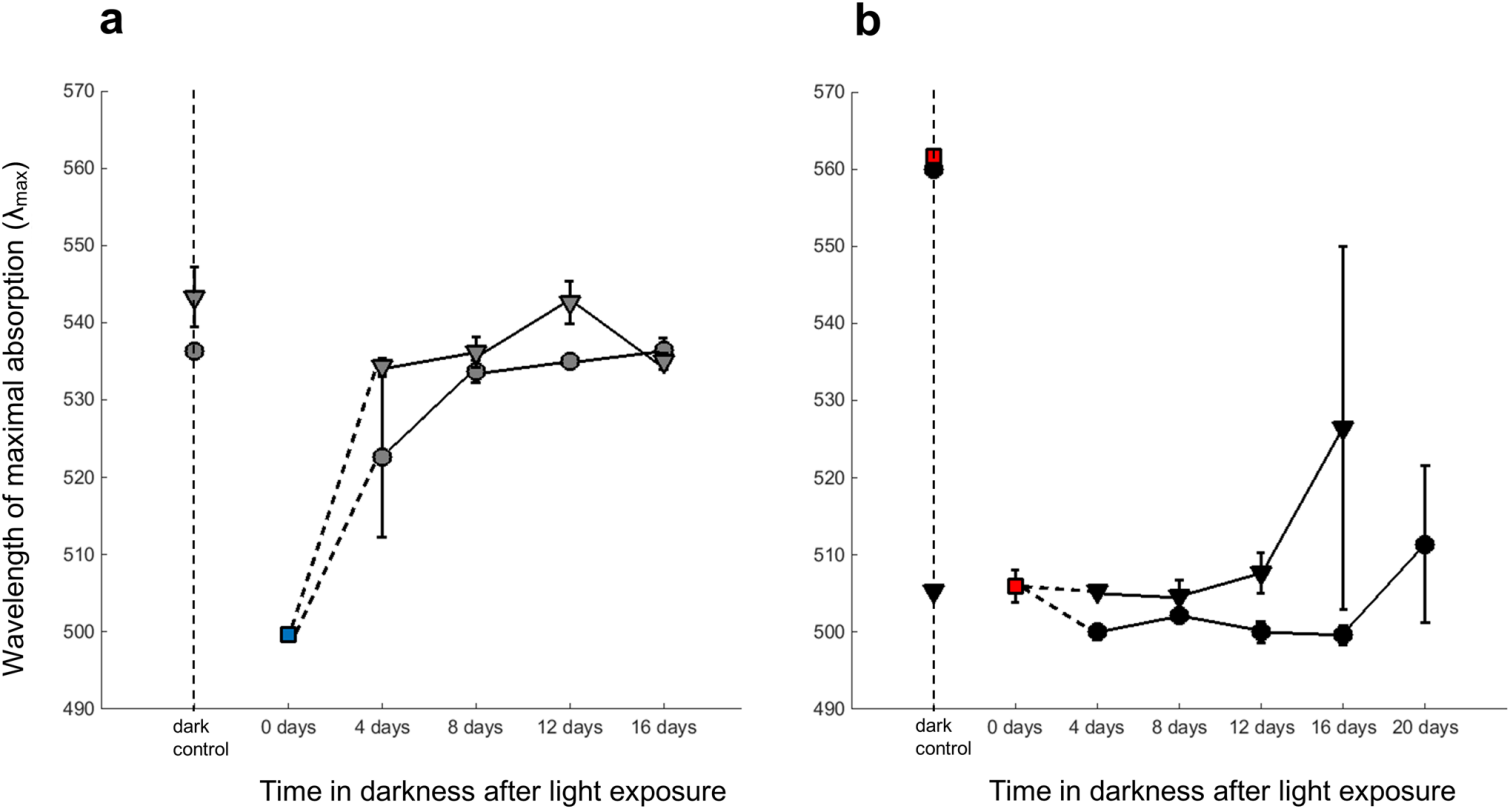
Wavelengths of maximum absorption (*λ*_max_) of spectra recorded by MSP from single rhabdoms of S_p_ animals (**a**), and L_p_ animals (**b**) kept in dark aquaria for different times after the standard light exposure. “Control” refers to animals had been kept continuously in darkness after capture (see "Materials and methods"). Data points and error bars give means ± SEM from measurements on 2–9 animals (measurements on 15–25 rhabdoms averaged within each individual). Triangles and circles refer to two independent sets of experiments done in different years. The blue square in (**a**) and the red squares in (**b**) mark values taken from Viljanen et al. (2017) to compensate for data points that were not available from the present experiments (see text). Note that the cohort marked by triangles in panel b, where the “controls” indicated earlier exposure to light, also had a head start in recovery

Illumination of a dark-adapted eye shifts the absorption spectrum of rhabdoms to shorter wavelengths, as the native rhodopsins (R), in mixtures that produce *λ*_max_ ≈ 535–560 nm in dark-adapted animals, are converted into metarhodopsins (MII) absorbing maximally around 490–500 nm (Viljanen et al. 2017). Restoration of native rhodopsin in the photoreceptor membranes can then be monitored by MSP as a shift of absorption spectra back towards longer wavelengths. Figure 5 plots changes of *λ*_max_ measured in rhabdoms from non-exposed (dark-adapted) controls and from animals that had spent different times in darkness after the light exposure (panel a for S_p_, panel b for L_p_). Each panel shows mean *λ*_max_ ± SEM of spectra averaged within individuals, circles, and triangles marking two different sets of experiments, performed in different years. First, consider S_p_ (panel a). The *λ*_max_ values of the non-exposed controls do not differ significantly from the mean ± SEM reported by Donner et al. (2016) for dark-adapted animals of the same population (#11 in their Table 1), 535.0 ± 2.0 nm. This was attributed to combined absorption by two rhodopsins with *λ*_max_ ≈ 525 and 570 nm. The light exposure shifted *λ*_max_ to shorter wavelengths, consistent with R → MII conversion. The immediate post-exposure value (0 days: *λ*_max_ ≈ 500 nm, blue square) is here taken from Viljanen et al. (2017); their Fig. 5), because in the present set of experiments, it was not possible to do MSP immediately after the exposure (see "Materials and methods"). In S_p_, *λ*_max_ increased rather quickly, reaching the dark-adapted control level after 12 days of dark-adaptation. A comparison with the regeneration of 11-*cis* retinal (Table 2 and grey bars in Fig. 2c) suggests that 60–70% of the original “dark” amount of 11-*cis* retinal was enough to support full restoration of native rhodopsin in the microvillar membranes.

Figure 5b shows corresponding data from L_p_ animals. Again, the *λ*_max_ values of dark-adapted controls (non-exposed animals: red square and black circle top left) are consistent with the value (561.0 ± 0.3 nm) reported by Donner et al. (2016) for the same population (#10 in their Table 1), and the light exposure shifted *λ*_max_ to wavelengths around 500 nm as expected from R → MII conversion. The immediate post-exposure point (0 days, red square) is again taken from Viljanen et al. (2017) to substitute for missing values. Another flaw in Fig. 5b is that the presumed “dark” control in one of the two sets of experiments (the leftmost triangle) is from animals that had not been thoroughly dark-adapted after capture, and *λ*_max_ ≈ 505 nm is indicative of light-exposed rhabdoms. To compensate for this, another “dark” value (red square at top left) is reproduced from Viljanen et al. (2017).

**Fig. 6:**
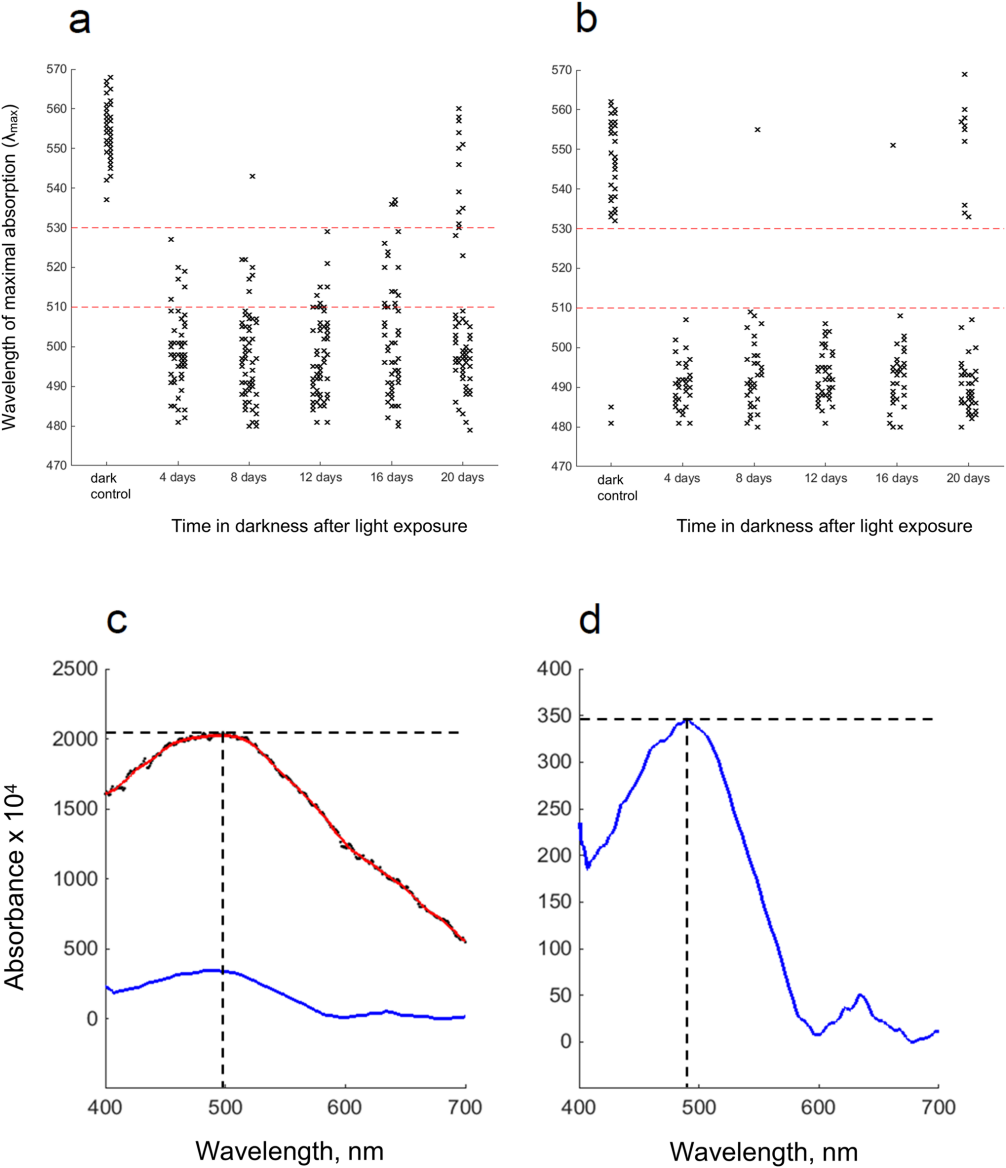
**a**, **b** Distribution of *λ*_max_ values of single L_p_ rhabdoms recorded at the respective time points in the experiment marked by circles in Fig. 5b. The two panels differ in the method of determination of *λ*_max_, as illustrated in panels c and d (see "Materials and methods"). **a** *λ*_max_ read automatically in Matlab from the peak of averaged, smoothed spectra recorded with T polarization. An example of this is shown in (**c**). Original data are shown by black dots and the smoothed spectrum by the red curve. The dashed lines indicate *λ*_max_. **b**: *λ*_max_ read automatically in Matlab from the peak of T–L difference spectra. In the example in (**d**), the blue curve is the smoothed T–L difference spectrum from the same rhabdom as in (**c**) (rescaled from the blue curve in (**c**)). This purifies absorbance by visual pigment molecules sitting in reasonably well-ordered microvilli, because other sources of absorbance will not exhibit pronounced dichroism. T–L difference spectra generally give somewhat lower *λ*_max_ values than the full spectra

Otherwise, the results are clear and present a very different picture from S_p_. The mean *λ*_max_ of L_p_ rhabdoms did not increase at all for 12–16 days post-bleach, and after that only weakly and with huge dispersion (error bars). This called for a closer look at the distribution of values from single rhabdoms. In Fig. 6 (data from the experiment marked by circles in Fig. 5b), panel a shows *λ*_max_ values determined automatically from all single-rhabdom T spectra of acceptable quality (cf. panel c: Method 2 described in "Materials and methods"). In the dark control, all values fell in the range 535–570 nm consistent with mixtures of two native rhodopsins at *λ*_max_ ≈ 525 and 570 nm (see above). After the light exposure, the great majority of spectra remained below 510 nm for up to 16 days, suggesting that most of the absorbance was due to MII, with only a few spectra at higher *λ*_max_. Not until 20 days did there emerge a subpopulation with *λ*_max_ > 530 nm. This dichotomy becomes even clearer in an analysis of T-L difference spectra, shown in panel b, which isolate absorption due to visual pigments in reasonably well-ordered photoreceptor membranes from “diffuse” absorption (Method 3 in "Materials and methods"). Panels c and d illustrate how the *λ*_max_ values in panels a and b have been obtained. These examples are representative also in showing that T-L difference spectra tend to give somewhat lower *λ*_max_ than the original spectra.

At present, we have no evidence that would allow us to associate the dichotomy (regenerating vs. non-regenerating rhabdoms) with specific regions of the eye or subpopulations of ommatidia reacting differently to strong light exposures. On the other hand, it is difficult to understand how such a clearly bimodal pattern would arise just from small random differences in the light exposure or the physiological state of the photoreceptors.

The presence of polarization sensitivity indicates that the photoreceptive membranes of the inert rhabdoms are not degraded, but retain integrity and significant microvillar orientation, and that the absorbance around 500 nm is indeed due to MII pigment with bound all-*trans* chromophore over the entire 20-day period. The higher level of all-*trans* retinal measured in L_p_ compared with S_p_ animals (Table 2) over post-exposure days 4–15 probably reflects this reserve of MII-bound chromophore.

### Recovery of light responsiveness of the eye

**Fig. 7:**
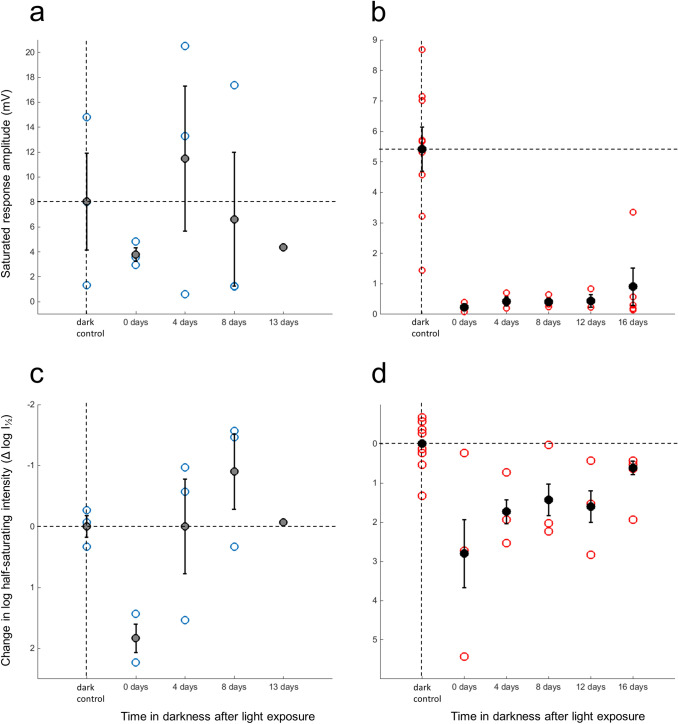
Measures of light responsiveness derived from response-amplitude vs. stimulus-intensity ERG recordings from eyes of dark-adapted, non-exposed animals (controls) and from animals that had spent different times in darkness after the standard light exposure. Left-hand panels (**a** and **c**): population S_p_, right-hand panels (**b** and **d**): population L_p_. Top panels: saturated response amplitude (*U*_max_, mV), bottom panels: log half-saturating stimulus intensity (log *I*_½_) expressed relative to dark-adapted controls, for which log *I*_½_ is set = 0. Note that fractional sensitivity is inversely proportional to *I*_½_, which is why log *I*_½_ is plotted on an inverted scale, so that sensitivity increases upwards and decreases downwards. The parameters were determined by fitting of Eq. 2 to the data. Filled circles and error bars show means ± SEM. The open circles (S_p_ blue and L_p_ red) show data from single experiments; all points are from different animals. Dark-adapted control values are indicated by the dashed lines drawn to facilitate the visual assessment of the post-exposure changes

The ultimately important biological question is how the processes considered above support the task of the eye, to enable vision. Visual function was measured by ERG from whole eyes attached to excised heads. Establishing changes in eye light responsiveness as a function of post-exposure adaptation history over days and weeks is challenging, as only one time point can be obtained from each preparation (animal), and unexplained variation between preparations is large. This arises from a number of factors: variation in eye optics, including the position of screening pigments and other factors affecting optical sensitivity (see, e.g., Frederiksen and Warrant 2008), variation in electrode penetration, affecting the geometry of current flow and resistivity in the extracellular space, on which the ERG field potential depends (cf. Donner et al. 1992), and variation in physiological factors unrelated to adaptation history. This should be kept in mind when judging the dispersion of single data points (coloured open circles) in Fig. 7.

The four panels a–d display, for both populations, the two main parameters used to characterize light responses (see Eq. 2). The top panels (a and b) show the maximum response amplitude (*U*_max_, mV), the bottom panels (c and d), and (logarithm of) the light intensity *I*_½_ needed to elicit a half-maximal response. Log *I*_½_ is displayed relative to the dark-adapted value (set = 0) on inverted ordinates, so that fractional sensitivity *S* (*S* ∝ *I*_½_^−1^) increases upwards and decreases downwards. The left-hand panels (a and c) show results for S_p_ animals, the right-hand panels (b and d) show the results for L_p_ animals. Means ± SEMs are marked by solid symbols and error bars, data from single experiments by open circles (blue for S_p_, red for L_p_). Note that no two *U*_max_ data points and no two *I*_½_ data points are from the same animal.

For S_p_ animals, the only reliable observation is that fractional sensitivity (panel c) dropped sharply immediately after the light exposure (time 0 days), by some 2 log units compared with the dark-adapted control animals. There is also a hint of depression of *U*_max_ at 0 days, consistent with earlier results of Viljanen et al. (2017). Beyond that, little can be said with confidence, but the impression is that at times ≥ 4 days post-exposure, neither *U*_max_ nor *I*_½_ was different from dark controls.

In L_p_ animals, the light exposure caused large and consistent changes in *U*_max_ (Fig. 7b), with a dramatic drop immediately after the light exposure and hardly any recovery over 12 days in darkness. Up to that point, the maximal response amplitude of all the light-exposed animals stayed below the entire variation range found in dark-adapted animals, and only on day 16 were there any signs of recovery. The general impression is strikingly similar to the time course of rhodopsin restoration in L_p_ rhabdoms (Figs. 5b, 6).

Changes in log *I*_½_ followed a similar pattern, although due to the large variation, it would be possible to demonstrate statistically significant differences only if all post-exposure data were pooled and tested against the dark-adapted controls. It should be remembered, however, that, e.g., 2-log-unit increases in *I*_½_ mean that 100-fold higher light intensities are needed to produce half of response amplitudes *U*_max_ that are already in themselves severely depressed. Thus, absolute sensitivities (mV/photoisomerization) are extremely low over the whole post-exposure period from 0 to 16 days.

It should finally be noted that pigment migration is unlikely to play any significant role as an adaptation mechanism in these experiments, because the screening pigments of the eyes are always in an essentially light-adapted position in the isolated-head preparation (Jokela-Määttä et al., 2005; Demchuk et al. 2012).

## Discussion

The current picture of the visual cycle in insects and crustaceans (“pancrustacea”: Regier et al. 2005) (Fig. 1) shows striking similarities to that in vertebrates (cf. Arshavsky 2010), although some features are likely to reflect evolutionary convergence rather than common origin (Srivastava and Goldsmith 1997). A fairly recent unifying realization was that an enzymatic retinoid cycle exists even in diurnal insects (Wang et al. 2010, 2012a, b; Montell 2012), long thought to rely wholly on photoregeneration (Hamdorf 1979). Within this general framework, however, details and weights of different elements vary greatly depending on both phylogeny and ecology. Insects differ from crustaceans (Srivastava et al. 1996), and diurnal from nocturnal species (e.g., mantis shrimps: Goldsmith and Cronin 1993). The present work is concerned with differences between two populations that differ minimally in both genetics (belonging to the same species) and ecology (living in low-light aquatic habitats). Still, they differ significantly in their recovery from strong light exposures, eyes of the “lake” population L_p_ being much more susceptible to long-term depression of visual function than those of the “sea” population S_p_ (Lindström and Nilsson 1988; Lindström et al. 1988; Viljanen et al. 2017). The central aim of the present study was to identify differences in the visual cycle that would correlate with this overall difference.

### The retinoid cycle

*Retinals*. Changes in isomers of retinal, retinol, and retinyl esters isomers over 2–3 weeks after a brief exposure of dark-adapted animals to strong white light were largely consistent with the previous work on crabs and crayfish. Illumination led to sharp decreases in 11-*cis* retinal and increases in all-*trans* retinal, but in both populations, the all-*trans* increase amounted to only ca. 20% of the 11-*cis* decrease. This indicates quick removal of all-*trans* retinal by reduction to all-*trans* retinol, which is a universal feature of retinoid cycling in animal eyes (e.g. Smith and Goldsmith 1991; Ala-Laurila et al. 2006; Wang et al. 2010, 2012a, b). Of course, it is in the alcohol form that the chromophore enters the regeneration cycle (Fig. 1), but fast removal of all-*trans* retinal may be desirable in itself, because the aldehyde is toxic (Różanowska and Sarna 2005; Maeda et al. 2009; Wang et al. 2012a, b). One notable difference between the two populations was the consistently higher levels of all-*trans* retinal in L_p_ than in S_p_ animals from post-exposure day 4 all the way to day 15 (Table 2). The likely origin is the large reserve of all-*trans* retinal that remains bound to inert MII in the membranes of L_p_ rhabdoms (see Fig. 6 and below).

At the functional end point of the retinoid cycle, regeneration of 11-*cis* retinal was somewhat faster in S_p_ than in L_p_, but the difference was not dramatic (Fig. 2c).

*Retinols*. Light absorption by the hexane extracts around the retinoid peak (Fig. 3) was much higher in L_p_ compared with S_p_ animals (in darkness ca. sevenfold measured at 325 nm). This is qualitatively consistent with the results of Feldman et al. (2010), who attributed 40% of the retinoid peak to retinol. The light exposure caused further massive release of free retinols in L_p_. In the set of HPLC measurements summarized in Table 3, total free retinol increased by sixfold between dark-adapted controls and post-exposure day 0 (ca. 50 → 300 units), and by as much as tenfold (ca. 40 → 400 units) in a previous set of similar experiments. As seen in Table 2, the decrease total retinal was very much smaller, only by a factor of 1.8 (27 → 15 arbitrary units) between controls and post-exposure day 0. This means that the retinol increase cannot be accounted for by reduction of light-released retinal, but must be derived from a pre-existing reserve, presumably retinyl esters (Wald 1957; Suzuki 1988; Goldsmith and Cronin 1993; Srivastava et al. 1996).

It is worth noting that the retinol increase involved both of the main isomers: 11-*cis* increased by as much as 7.5-fold, all-*trans* by fourfold (Table 3). How may these be derived from 11-*cis* and all-*trans* retinyl esters? Srivastava et al. (1996), studying lobster and crayfish (dim-light macruran decapods like *Mysis relicta*), suggested that all*-trans* retinyl esters form 11*-cis* retinol in the dark by a process similar to that in the vertebrate pigment epithelium. In our experiments, however, 11-*cis* retinol was low in the dark-adapted controls, and its increase after the light exposure correlated closely with a decrease in 11-*cis* retinyl esters (Fig. 4 and Tables 3, 4), suggesting that the mechanism described by Srivastava et al. (1996) does not work in *Mysis*. It seems likely that all*-trans* retinyl esters release all-*trans* retinol without enzymatic dark isomerization into the 11-*cis* form. In any case, it is worth noting that immediately after the light exposure, total retinols + retinyl esters jumped by 75% to a higher level (controls vs. day 0, summed from Tables 3, 4), which was then maintained over the 12 days of the experiment. This suggests that there was recruitment from stores outside the small part of the head from which samples were prepared. The duration of the light exposure, 30 min, leaves time for significant transport to occur before the illuminated animal is sacrificed. This underscores a more general problem of the present experimental design: studying dark-adaptation processes occurring in intact living animals in samples prepared from small pieces of head does not allow quantitative analysis of retinoid metabolism as a closed cycle.

During the subsequent dark-adaptation, the opposite tendency was observed, where decreases in free retinols correlated with increases in 11-*cis* retinyl esters and 11-*cis* retinal. After about 15 days in darkness the levels of all the studied retinoids reached values similar to those of animals kept in darkness for 6–8 weeks.

*The L*_*p*_*/S*_*p*_* retinol difference*. The major difference in the retinoid cycle of L_p_ and S_p_ animals is the much higher level of the retinol budget in L_p_, both in darkness and in terms of light-induced release (Fig. 3). Functional interpretation inevitably remains speculative. Feldman et al. (2010) suggested that animals inhabiting extremely dim-light environments (L_p_), where no photoregeneration MII → R can occur, need a large store of chromophore (or its precursors) for effective ‘‘dark’’ regeneration of visual pigment. This remains a possible partial interpretation, but does not explain the great excess of retinols compared with the dynamics of changes in total retinal. It has to be remembered, however, that the amounts reported here were measured from animals that had spent 6–8 weeks in complete darkness, had then been exposed to a strong white light that they would never encounter in their natural habitats, and had finally been left in complete darkness again. The massive retinol release could be an inappropriate response of a sensitive recruitment system, tuned to react to illumination changes of a few tens rather than thousands of photons per second impinging on the eye. With respect to the difference in basal retinol levels between L_p_ and S_p_, it is worth noting that the rhabdoms of dark-adapted S_p_ show a higher degree of microvillar disorder than those of dark-adapted L_p_ (Viljanen et al. 2017). This suggests that S_p_ has an intrinsically higher rate of membrane and pigment turnover even in darkness (conceivably associated with general acclimation to somewhat higher light levels), which could keep the steady-state level of retinoid stores lower. Conversely, low intrinsic rates of membrane cycling in L_p_ could lead to build-up of higher steady-state retinol levels. Whether these high levels may, in turn, contribute to other effects of strong light exposures (see below) can only be the subject of speculation at this point.

### Restoration of native rhodopsin in the photoreceptors and recovery of eye light responses

Changes in the R:MII ratio in the rhabdomal membranes were monitored through shifts of the absorbance spectra. Viljanen et al. (2017), who applied the same standard light exposure as used in the present work to dark-adapted S_p_ and L_p_ animals, found that *λ*_max_ immediately dropped to levels consistent with MII dominance, around 500 nm. Here, we measured spectral changes after that in rhabdoms from animals left to dark-adapt and sampled at time points from 4 days up to 16 or 20 days after the exposure. Strict quantitative analysis of pigment proportions is not possible, since single-rhabdom spectra in both populations may arise from contributions, in different ratios, from two rhodopsins (tentative *λ*_max_ ≈ 525 and 570 nm: Donner et al. 2016), and their metarhodopsins (*λ*_max_ ≈ 490–500 nm). It should also be noted that MSP sampling is not truly random, as rhabdoms with good morphology and little or no screening pigment on them are more likely to be selected for measurement and further analysis. For our present purpose, however, it was sufficient to follow the general return of the composite spectra towards the *λ*_max_ of the dark-adapted pre-exposure controls.

The return kinetics of S_p_ *λ*_max_ (Fig. 5a) was consistent with the kinetics of regeneration of 11-*cis* retinal if one accepts that 60–70% of the original 11-*cis* suffices to restore the full complement of native rhodopsin. The admittedly crude ERG data (Fig. 7a, c) at least do not indicate the presence of any extra delay between rhodopsin restoration and the recovery of light responses.

The very different time course of *λ*_max_ changes in L_p_ rhabdoms (Fig. 5b), involving 12–16 days of near-stasis after the light exposure, is parallelled by a similarly long-lasting suppression of ERG light responses (Fig. 7b). These ERG measurements extend previous results by Lindström et al. (1988) who found that light responses of L_p_ animals that had been subjected to a similar (possibly somewhat weaker) exposure “were approaching normality approximately 100 h post-exposure”. The present results on L_p_, as well as S_p_, suggest that recovery of eye light sensitivity closely follows the restoration of native rhodopsin in rhabdoms.

Scrutiny of the distribution of single-rhabdom absorption spectra (especially T–L difference spectra) in L_p_ (Fig. 6a, b) yielded several important insights.

First, most rhabdoms showed no sign of incorporating new pigment, but remained inert over the entire 20-day period. The incipient recovery of mean *λ*_max_ towards values typical of the native rhodopsins depended wholly on a minority of rhabdoms. On the other hand, these rhabdoms appeared to be nearly fully recovered after 20 days.

Second, the fact that the MII-type spectra of the inert rhabdoms showed T-L dichroism indicates that the metarhodopsin was sitting in microvillar membranes that retained a significant degree of order. Judging by the ERG recordings, this (probably arrestin-bound) MII was unable to activate phototransduction (cf. Kiselev and Subramaniam 1994).

Third, the apparently complete lack of incorporation of new rhodopsin in the “silent majority” of rhabdoms indicates that incorporation of new native pigment occurs only through membrane turnover, as suggested by Cronin and Goldsmith (1993). The results of Viljanen et al. (2017), indicating higher degrees of microvillar disorganization in dark-adapted S_p_ compared with dark-adapted L_p_ rhabdoms, support the conclusion that the central factor in the compromised sensitivity of L_p_ eyes is not the disruption of membranes or microvillar organization, but the stasis in membrane renewal, which leaves most membranes occupied by inert MII. Our general conclusion is that sensitivity recovery of S_p_ eyes after a strong light exposure is rate-limited by the regeneration of 11-*cis* retinal, whilst that of L_p_ eyes is limited by inertia in photoreceptor membrane turnover.

## Abbreviations

A1: Chromophore A1 (retinal), or visual pigment using that chromophore
A2: Chromophore A2 (3,4,-didehydroretinal) or visual pigment using that chromophore
*C*_i_: Amount of retinal, retinol, or retinyl ester isomers in sample *i*, in arbitrary units proportional to absolute quantities
ERG: Electroretinography
HPLC: High-performance liquid chromatography
*I*_½_: Half-saturating intensity: the stimulus light intensity that elicits 50% of the saturating response amplitude *U*_max_ in an ERG experiment
IR: Infra-red
L: Longitudinal polarization: light linearly polarized along the long axis of the rhabdom
*λ*_max_: The wavelength of maximum absorption of a visual pigment, receptor cell, or whole rhabdom
L_p_: The population of *Mysis relicta* inhabiting Lake Pääjärvi (“Lake”), or an individual from that population
MII: Metarhodopsin II
MSP: Microspectrophotometry
R: Rhodopsin in its native configuration bound to 11-*cis* retinal
RPE: Retinal pigment epithelium
*S*: Fractional sensitivity of the eye: response amplitude per photoisomerization expressed as a fraction of the saturating response amplitude *U*_max_ in an ERG experiment. *S* is inversely proportional to *I*_½_
S_p_: The population of *Mysis relicta* inhabiting Pojoviken Bay of the Baltic Sea (“Sea”), or an individual from that population
T: Transverse polarization: light linearly polarized orthogonally to the long axis of the rhabdom
*U*_max_: Saturating (maximal) response amplitude of the eye in an ERG experiment
